# MicroRNAs Up-Regulated by CagA of *Helicobacter pylori* Induce Intestinal Metaplasia of Gastric Epithelial Cells

**DOI:** 10.1371/journal.pone.0035147

**Published:** 2012-04-20

**Authors:** Yongliang Zhu, Qiaoli Jiang, Xiaojun Lou, Xiaowei Ji, Zhenzhen Wen, Jia Wu, Haiying Tao, Tingting Jiang, Wei He, Caihua Wang, Qin Du, Shu Zheng, Jianshan Mao, Jian Huang

**Affiliations:** 1 Department of Gastroenterology, Second Affiliated Hospital of Zhejiang University School of Medicine, Hangzhou, Zhejiang Province, China; 2 Cancer Institute and Education Ministry Key Laboratory of Cancer Prevention and Intervention, Zhejiang University School of Medicine, Hangzhou, Zhejiang Province, China; Veterans Affairs Medical Center (111D), United States of America

## Abstract

CagA of *Helicobacter pylori* is a bacterium-derived oncogenic protein closely associated with the development of gastric cancers. MicroRNAs (miRNAs) are a class of widespread non-coding RNAs, many of which are involved in cell growth, cell differentiation and tumorigenesis. The relationship between CagA protein and miRNAs is unclear. Using mammalian miRNA profile microarrays, we found that miRNA-584 and miRNA-1290 expression was up-regulated in CagA-transformed cells, miRNA-1290 was up-regulated in an Erk1/2-dependent manner, and miRNA-584 was activated by NF-κB. miRNA-584 sustained Erk1/2 activities through inhibition of PPP2a activities, and miRNA-1290 activated NF-κB by knockdown of NKRF. *Foxa*1 was revealed to be an important target of miRNA-584 and miRNA-1290. Knockdown of *Foxa*1 promoted the epithelial-mesenchymal transition significantly. Overexpression of miRNA-584 and miRNA-1290 induced intestinal metaplasia of gastric epithelial cells in knock-in mice. These results indicate that miRNA-584 and miRNA-1290 interfere with cell differentiation and remodel the tissues. Thus, the miRNA pathway is a new pathogenic mechanism of CagA.

## Introduction

*Helicobacter pylori* (*H*.*pylori*) infection is one of the main inducers of chronic active gastritis and peptic ulcer; it is also closely associated with the genesis and development of gastric cancer and gastric mucosa–associated lymphoid tissue (MALT) lymphoma. The Union for International Cancer Control has documented *H*.*pylori* as a type I carcinogen [1]–[3]. China has among the highest incidence rates of both *H*.*pylori* infection and gastric cancer, and the *H*.*pylori* strain positive for the virulence factor *cagA* (*cagA*+ *H*.*pylori*) is the most common type [4]. CagA plays important roles in the genesis and development of precancerous gastric lesions, gastric cancer, and MALT lymphoma, which indicates that CagA is a bacterium-derived tumor-associated protein [5]–[7]. CagA can destroy the apical tight junction complex of MDCK cells in a non–phosphorylation-dependent manner to damage cell barriers, leading to “leakage” between gastric mucosal epithelial cells [8]. After injection into host cells by the type IV secretion system, CagA is phosphorylated on tyrosine residues by c-Src and Lyn kinases. Phosphorylated CagA then associates with and activates a bona fide oncoprotein, SHP-2, leading to Erk1/2 pathway activation. F-actin stress fibers become highly polarized, which leads to cytoskeleton reorganization and cell dissociation into a “hummingbird”-like phenotype [9]–[11]. In previous study, we had found that activation of the Erk1/2 kinases by CagA directly induces immortalized NIE epithelial cell transformation, and the proliferation activity of transformed cells is significantly inhibited after Erk1/2 inhibition [12]. Ohnishi *et al* showed that *cagA*-transgenic mice develop gastric cancer and MALT lymphoma at 72 weeks [13]. Saadat *et al* showed that CagA causes abnormal chromatin segregation during mitosis through SHP-2 activation of microtubule affinity-regulating kinase [14].

MicroRNAs (miRNAs) are a class of widely distributed, non-coding, single- stranded RNAs composed of about 19 to 22 nucleotides. In mammalian cells, miRNAs bind to 3′ untranslated regions (UTR) of mRNAs mainly through incomplete base- pairing to inhibit gene translation at the post-transcriptional level and thereby down-regulate the expression of target genes [15]. miRNAs regulate cell growth, differentiation, stress, and many other biological processes [16]–[21]. Previous studies have found that some miRNAs could be altered after *H*.*pylori* infection [22], [23]. However, whether cagA is involved in cellular regulation of certain miRNAs in the gastric epithelium remains elusive.

Intestinal metaplasia is a precancerous lesion of the stomach in which there is transdifferentiation of the gastric mucosa to an intestinal phenotype. Intestinal metaplasia of the gastric antrum is common in adults with *H*.*pylori*–associated chronic gastritis. As we know that intestinal meteplasia is associated with *H*.*pylori* infection, eradication of *H*. *pylori* infection could significantly attenuate this condition [24]. An intestine-specific transcription factor, CDX2, is involved in the induction of intestinal metaplasia in the stomach [25]. Whether is there is another molecular mechanism to induce intestinal metaplasia besides CDX2 is still unknown. Therefore, we wanted to know whether the tumorigenic CagA protein also affects the expression of miRNAs to induce intestinal metaplasia of gastric mucosa.

Here, we found that both miRNA-584 and miRNA-1290 were up-regulated by CagA. Overexpression of these miRNAs induced intestinal metaplasia in knock-in mice. These results indicate that the miRNA pathway is a new pathogenic mechanism of CagA.

## Results

### 1. Up-regulation of miRNA-584 and miRNA-1290 Expression by CagA Protein

**Figure 1:**
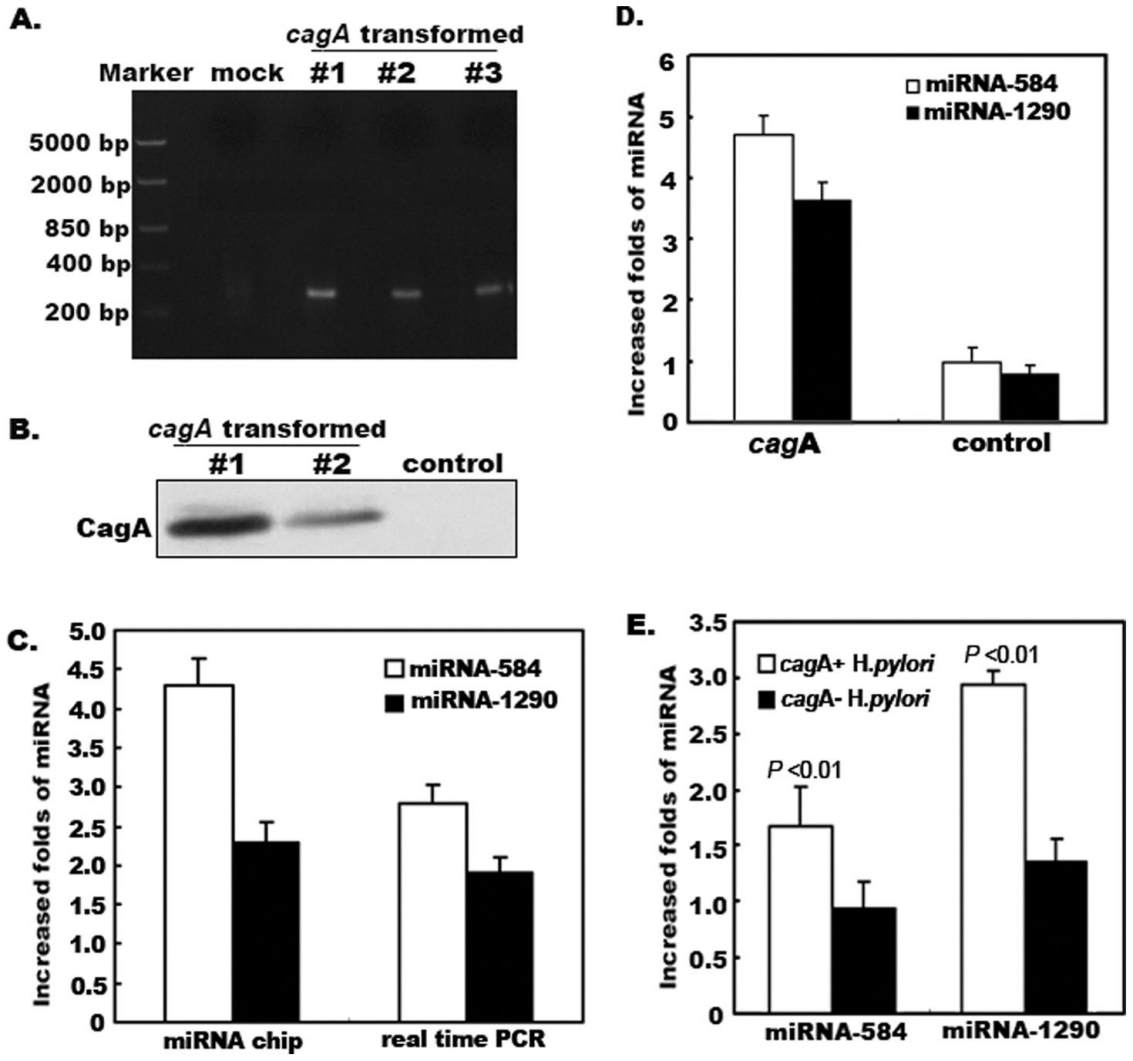
Screening and identification of miRNA-584 and miRNA-1290 in CagA-transfected cells. A. Detection of *cagA* DNA fragments in transfected cells. The standard calcium phosphate precipitation method was used to establish stable CagA-expressing gastric carcinoma AGS cells. Transfection was validated using PCR and DNA sequencing after cell clones had grown. AGS cells stably expressing the empty *pEGFP-c*1 vector were also established as a control. **B. Detection of CagA protein in transfected cells.** Stable CagA-expressing gastric carcinoma AGS cells were lysed with RIPA buffer. The lysates were subjected to SDS-PAGE and subsequently to western blot analysis to probe with anti-GFP antibodies. AGS cells stably expressing the empty *pEGFP-c*1 vector were also established as a control. **C. Screening and identification of miRNA-84 and miRNA-290 in stable CagA-expressing AGS cells.** Total RNA was extracted from stable CagA-expressing AGS cells and control cells. Mammalian miRNA expression profile microarray scanning and TaqMan real-time PCR were performed. The results showed that both miRNA-584 and miRNA-1290 were up-regulated in stable CagA-expressing AGS cells. **D. Identification of miRNA-584 and miRNA-1290 in gastric carcinoma SGC7901 cells transiently expressing CagA.** Total RNA from gastric carcinoma SGC7901 cells transfected with *cagA*/*pEGFP-c*1 plasmid was extracted and real-time PCR was performed. Both miRNA-584 and miRNA-1290 were up-regulated in transiently CagA-expressing SGC7901 cells. **E. Identification of miRNA-584 and miRNA-1290 in gastric carcinoma AGS cells infected with** ***H*** **.** ***pylori*** **.** Total RNA was extracted from AGS cells infected with *H*.*pylori* including NCTC11637 and 9 *cagA*– isolates for 7 h (MOI≈50) respectively, and TaqMan real-time PCR was performed. Both miRNA-584 and miRNA-1290 were up-regulated after infection of *cagA*+ *H*.*pylori* (all *P* <0.01, ANOVA). Data are represented as mean +/− s.e.m.

The pathogenesis of CagA was induced by which was injected into epithelial cells by the type IV secretion system of *H*.*pylori*. To mimic injected CagA in the host cell, we first used the standard calcium phosphate precipitation method to establish stable CagA-expressing gastric carcinoma AGS cells to explore whether CagA protein affects miRNA expression (**Figure 1a and 1b**). We then screened for affected miRNAs in CagA-transformed gastric carcinoma AGS cells and control empty vector–expressing cells using mammalian miRNA expression profile microarrays. After scanning 1024 miRNAs, we found that the expression of miRNA-584 and miRNA-1290 was up-regulated in CagA-transformed cells, but no down-regulated miRNA was found. These results were further verified by TaqMan real-time PCR (**Figure 1c**). Moreover, both transient transfection of *cagA* into gastric carcinoma SGC7901 cells and NCTC11637 *H*.*pylori*–infected AGS cells for 7 h yielded similar results (**Figure 1d and 1e**). The above results indicate that CagA protein up-regulates miRNA-584 and miRNA-1290 expression and suggests that CagA may affect cellular functions by altering miRNA expression.

### 2. miRNA-1290 was Up-regulated in an Erk1/2-dependent Manner and miRNA-584 was Activated by NF-κB

**Figure 2:**
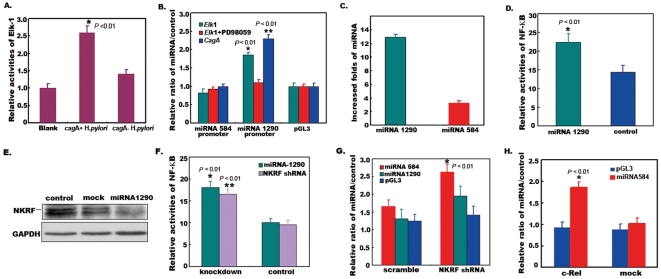
miRNA-1290 was up-regulated in an Erk1/2-dependent manner and miRNA-584 was activated indirectly by miRNA-1290. A. Determination of transactivities of Elk-1. AGS cells were transiently transfected with pGL3-Elk-1 plasmid for 24 h and subsequently infected with *H*.*pylori* including NCTC11637 and 7 *cagA*+ isolates for additional 7 h (MOI≈50) respectively. Cells were lysed and the lysates were collected for determination of luminescent intensities by dual-luciferase ÿreporter assay. The results showed that transactivities of Elk-1 was up-regulated after infection of *cagA*+ *H*.*pylori* (*P* <0.01, ANOVA). **B. Determination of luciferase activations containing promoter region of miRNA-584 and miRNA-1290.** Plasmids containing *Elk*-1 or *cagA* were co-transfected with recombinant luciferase reporters containing promoter regions of miRNA-584 and miRNA-1290 (≈2000 bp) respectively into 293T cells for 24 h (*Elk-*1/*cagA*: luciferase reporters: Renilla luciferase = 3∶1∶0.01). Cells were lysed and the lysates were collected for determination of luminescent intensities by dual-luciferase_reporter assay. The results showed that miRNA-1290 was up-regulated by both Elk-1 and CagA (*P* <0.01, ANOVA), whereas miRNA-584 was not affected. **C. Determination of miRNA-584 in stable miRNA-1290–expressing cells.** To determine miRNA-584 in stable miRNA-1290–expressing cells, total RNA from stable miRNA-1290–expressing cells were isolated and real-time PCR was performed for miRNA-584. The results showed that miRNA-584 was up-regulated in stable miRNA-1290–expressing cells. **D. Expression of miRNA-1290 enhanced NF-κB activities.** miRNA-1290 was co-transfected with NF-κB reporter into SGC7901 cells for 24 h (miRNA-1290: luciferase reporters: Renilla luciferase = 3∶1∶0.01). Cells were lysed and the lysates were collected for determination of luminescent intensities by dual- luciferase_reporter assay. The results showed that miRNA-1290 enhanced NF-κB activities (*P* <0.01, ANOVA). **E. Detection of NKRF protein in transfected cells.** miRNA-1290-expressing gastric carcinoma SGC7901 cells were lysed and subsequently were subjected to western blot analysis to probe with anti-NKRF antibodies. SGC7901 cells expressing the empty vector were also established as a control. **F. Knockdown of NKRF increased NF-κB activities.** *NKRF* shRNA plasmid (Santa Cruz) was co-transfected with NF-κB reporters into SGC7901 cells for 48 h (*NKRF* shRNA plasmid: NF-κB reporters: Renilla luciferase = 3∶1∶ 0.1). Cells were lysed and the lysates were collected for determination of luminescent intensities. The results showed that knockdown of *NKRF* increased NF-κB activities (*P* <0.01, ANOVA). **G. Activation of NF-κB upregulated miRNA-584.** *NKRF* shRNA plasmid was co-transfected with recombinant luciferase reporters containing promoter regions of miRNA-584 into SGC7901 cells for 48 h (*NKRF* shRNA plasmid: luciferase reporters: Renilla luciferase = 3∶1∶ 0.1). Cells were lysed and the lysates were collected for determination of luminescent intensities. The results showed that NF-κB up-regulated miRNA-584 (*P* <0.01, ANOVA). **H. Overexpression of c-Rel upregulated miRNA-584.** *c-Rel* plasmid was co-transfected with recombinant luciferase reporters containing promoter regions of miRNA-584 into SGC7901 cells for 48 h (*c-Rel* plasmid: luciferase reporters: Renilla luciferase = 3∶1∶ 0.1). Cells were lysed and the lysates were collected for determination of luminescent intensities. The results showed that Overexpression of c-Rel upregulated miRNA-584 (*P* <0.01, ANOVA). Data are represented as mean +/− s.e.m.

To explore the mechanism of miRNA-584 and miRNA-1290 activation, we examined the transcript factor binding sites in the promoter regions of miRNA-584 and miRNA-1290, and found that there were Elk-1 binding sites in both promoter regions (**Figure S1**). Elk-1 is one of the important substrates of Erk1/2 kinases. Phosphorylated Elk-1 activated by Erk1/2 kinases potentiates ternary complex formation with serum response element (SRE), serum response factor (SRF), and c-fos to enhance targets’ transcription. It was confirmed that CagA could activate Erk1/2 kinases by directly binding SHP-2 and phosphorylated Elk-1 [26]. We also observed that transactivities of Elk-1 were up-regulated after infection of *cagA*+ *H*.*pylori* (**Figure 2a**). To find out whether CagA up-regulated miRNA-584 and miRNA-1290 in an Erk1/2-dependent manner, we constructed luciferase reporters containing the promoter regions of miRNA-584 and miRNA-1290 respectively. We found that miRNA-1290 was significantly up-regulated by both Elk-1 and CagA in 293T cells co-transfected with recombinant luciferase reporters and *Elk-*1 or *cagA*, whereas miRNA-584 was not affected (**Figure 2b**), which implied that there were different mechanisms for miRNA-584 and miRNA-1290 activation. On the other hand, we examined the relationship between miRNA-584 and miRNA-1290 in clinic tumor tissue samples and stable miRNA-1290 –expressing cells. Interestingly, a strong relationship was found between miRNA-584 and miRNA-1290 in clinic tumor tissue samples (**Figure S2**), in accordance with miRNA-584 being up-regulated in stable miRNA-1290 –expressing cells (**Figure 2c**). This suggests that miRNA-584 may be regulated by miRNA-1290. To analyze the mechanism of miRNA-584 activation, we further checked the transcript factor binding sites in the promoter regions of miRNA-584 again besides Elk-1, and found that there were c-Rel binding sites in the promoter regions of miRNA-584 (**Figure S3**). Whether does miRNA-1290 activate the NF-κB pathway due to the weak activation of NF-κB by CagA? To this end, we checked the NF-κB activities by co-transfecting NF-κB reporters with miRNA-1290 plasmids into gastric carcinoma SGC7901 cells. We found that NF-κB activities were enhanced at 24 h after transfection of miRNA-1290 (**Figure 2d**). Western blot showed that NF-κB repressing factor (NKRF) was knockdown after overexpression of miRNA-1290 (**Figure 2e**). NF-κB activities increased after knockdown of *NKRF* by shRNA (**Figure 2f**). These results implied that miRNA-1290 activated NF-κB by knockdown of NKRF. Finally, we examined whether miRNA-584 was activated by NF-κB. We found that miRNA-584 was significantly up-regulated in SGC7901 cells co-transfected with recombinant luciferase reporters and *NKRF* shRNA or *c-Rel* plasmids (**Figure 2g and 2h**). Together, miRNA-1290 was up-regulated in an Erk1/2-dependent manner and miRNA-584 was activated indirectly by miRNA-1290.

### 3. *cagA*+ *H*.*pylori* Activated Erk1/2 Kinases and miRNA-584 Sustained Erk1/2 Activities through Inhibition of Protein Phosphatase 2a (PPP2a)

**Figure 3:**
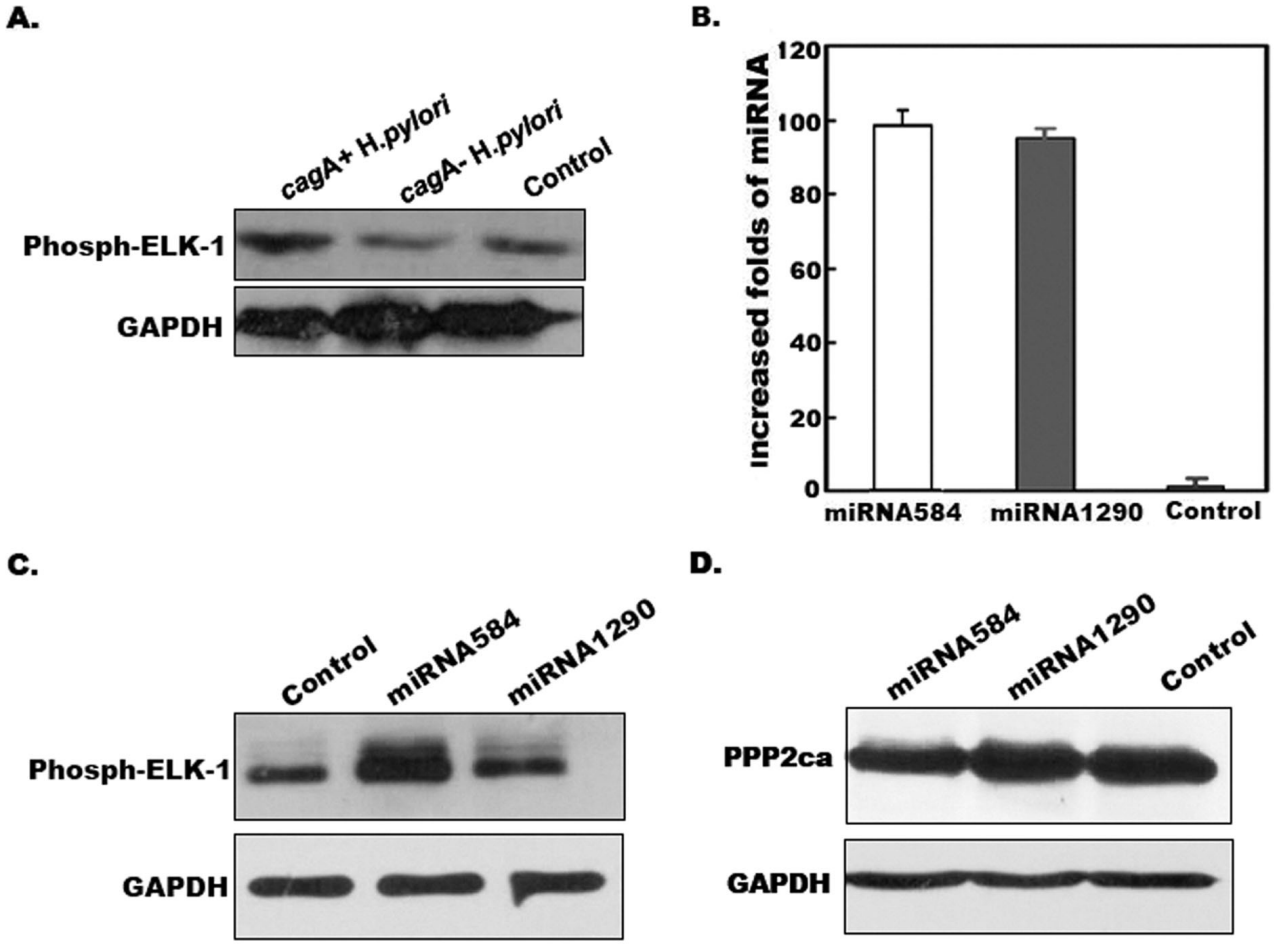
*cagA*+ *H*.*pylori* activated Erk1/2 kinases and miRNA-584 sustained Erk1/2 activities through inhibition of PPP2a. A. Erk1/2 kinases were activated by CagA. AGS cells were infected with *H*.*pylori* including NCTC11637 and 9 *cagA*– and 7 *cagA*+ isolates for 7 h (MOI≈50) respectively. Cells were collected and lysed at 24 h after transfection. The lysates were co-precipitated with agarose-conjugated anti-Erk1/2 antibodies, and after washing 5 times, the beads were mixed with recombinant Elk-1 and ATP. Mixtures were subjected to SDS-PAGE after denaturing and subsequently western blot analysis probed with antibodies against phosph-Elk-1. Uninfected AGS cells were also established as a control. **B. Determination of miRNA-584 and miRNA-1290 in transiently transfected cells.** Total RNA was extracted from gastric carcinoma AGS cells transiently transfected with plasmids containing miRNA-584 or miRNA-1290 at 24 h after transfection. Real-time PCR was performed for determination of miRNA-584 and miRNA-1290. **C. Detection of Erk1/2 activities.** Gastric carcinoma AGS cells were transiently transfected with plasmids containing miRNA-584 or miRNA-1290. Cells were collected and the lysates were co-precipitated with agarose-conjugated anti-Erk1/2 antibodies, and after washing 5 times, the beads were mixed with recombinant Elk-1 and ATP at 30°C for 30 min. Mixtures were subjected to SDS-PAGE after denaturing and subsequently western blot analysis probed with antibodies against phosph-Elk-1. The empty vector was also established as a control. **D. Detection of PPP2ca expression.** Gastric carcinoma SGC7901 cells were transiently transfected with plasmids containing miRNA-584 or miRNA-1290. Cells were collected and lysed at 24 h after transfection. Lysates were subjected to SDS-PAGE after denaturing and subsequently western blot analysis probed with antibodies against PPP2ca. Data are represented as mean +/− s.e.m.

We found that Erk1/2 kinases could be activated after infection of *cagA*+ *H*.*pylori* (**Figure 3a**). To clarify the effects of miRNA-584 and miRNA-1290 on Erk1/2 pathways, we evaluated the effects of miRNA-584 and miRNA-1290 on Erk1/2 signaling. Recombinant plasmids expressing mature miRNA-584 and miRNA-1290 were first constructed and transiently transfected into gastric carcinoma AGS cells (**Figure 3b**). Kinase assays and western blot analysis were used to detect Erk1/2 activities in transfected cells. Expressing miRNA-584 led to higher activation of Erk1/2, but miRNA-1290 had no significantly effect on Erk1/2 activation (**Figure 3c**). A TargetScan search found two potential binding sites for miRNA-584 in the 3′-untranslational region (UTR) of *PPP*2ca, one of the important phosphatases of phosphorylated Erk1/2 (**Figure S4**), Further, we found that PPP2a expression were inhibited in cells transfected with miRNA-584 (**Figure 3d**). These results suggest that miRNA-584 sustained Erk1/2 activities through inhibition of PPP2a activities.

### 4. *Foxa*1 is a Target of miRNA-584 and miRNA-1290 and Knockdown of *Foxa*1 Promotes Epithelial-mesenchymal Transition (EMT)

**Figure 4:**
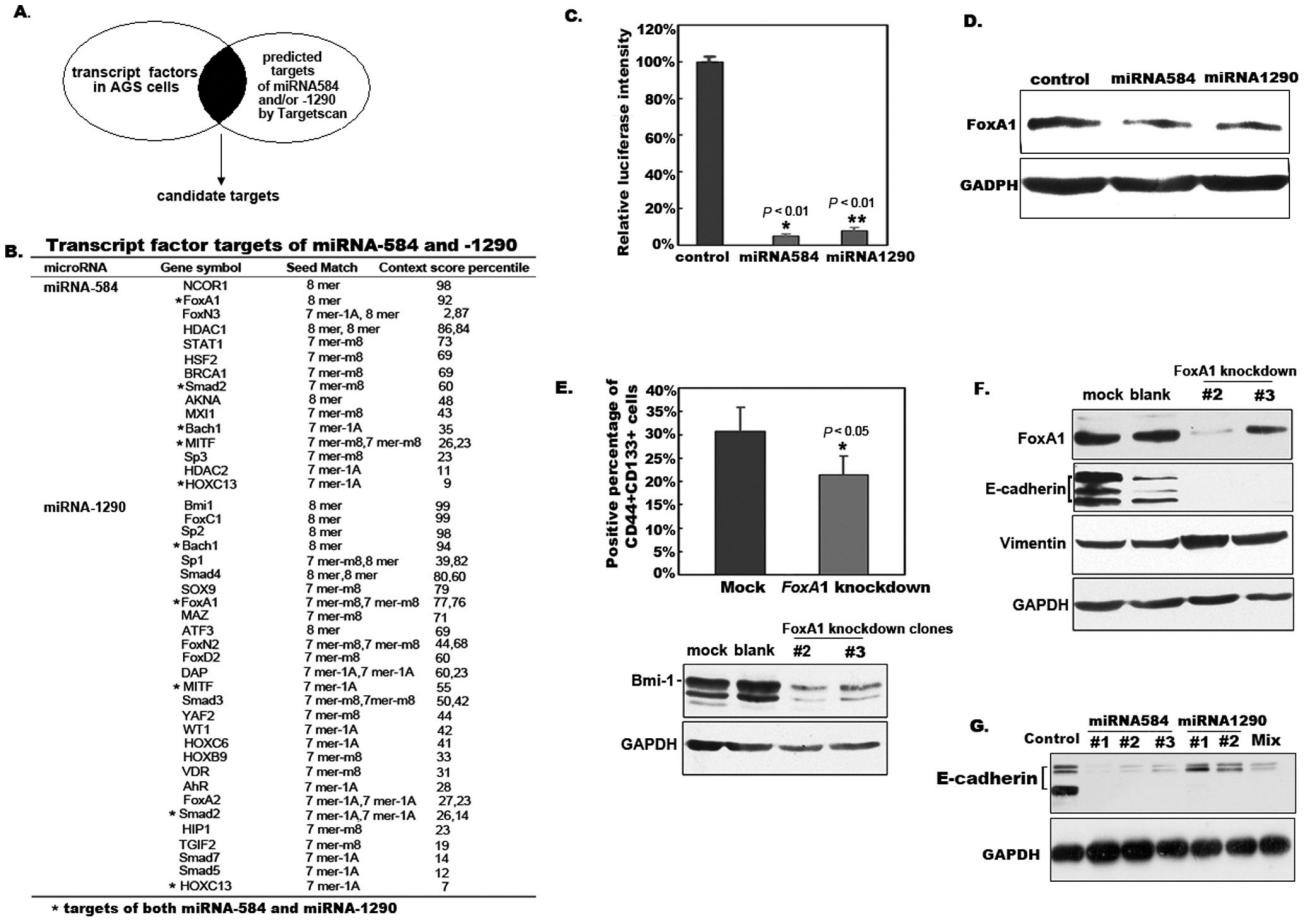
*Foxa*1 is a target of miRNA-584 and miRNA-1290 and knockdown of *Foxa*1 promotes EMT. A. Schematic of potential target screening strategy. B. Transcription factor targets of miRNA-584 and miRNA-1290. Transcription factors targeted by miRNA-584 and miRNA-1290 expressed in AGS cells were predicted by TargetScan (www. targetscan.org). **C. Determination of pMIR-report luciferase activity.** The recombinant plasmid pMIR-*Foxa*1 report was co-transfected with pcDNA6.2-GW/EmGFP-miR containing miRNA-584 or miRNA-1290 into AGS gastric cancer cells for 48 h. Cells were lysed and luciferase activity was determined by dual-luminescence reporter assay. Co-transfection of miRNA-584 or miRNA-1290 with pMIR-*Foxa*1 inhibited more than 90% of firefly luciferase activity (all *P* <0.001, ANOVA), indicating that miRNA-584 and miRNA-1290 target *Foxa*1 mRNA. **D. Detection of Foxa1 in cells transiently expressing miRNA-584 and miRNA-1290.** pcDNA6.2-GW/EmGFP-miR containing miRNA-584 or miRNA -1290 was transfected into SW620 colon cancer cells for 48 h. Cells (1×10^6^) were lysed and the supernatant was subjected to western blot analysis. The results showed that the expression of Foxa1 protein was down-regulated about 50% after transfection of miRNA-584 or miRNA-1290. These results indicate that Foxa1 is a target of miRNA-584 and miRNA-1290. **E. Analysis of the percentage of CD44^+^CD133^+^ cells.** SW620 colon cancer cells were infected with recombinant *Foxa*1 shRNA lentivirus. Infected SW620 cells were digested and resuspended at 1×10^6^ per 100 µL of PBS containing 2% FBS, and labeling antibodies (APC-CD44 and PE-CD133) were added at 4°C for 30 min in the dark. After two washes, cells were analyzed using a FACSAria. An empty vector control was also established. The percentage of CD44^+^CD133^+^ cells significantly decreased after *Foxa*1 knockdown (*P* <0.05, ANOVA). The supernatant from SW620 colon cancer cells with *Foxa*1 knockdown was subjected to western blot for detection of Bmi1. **F.** ***Foxa*** **1 knockdown promotes EMT.** The supernatant from SW620 colon cancer cells with *Foxa*1 knockdown was subjected to SDS-PAGE, and proteins were transferred onto a nitrocellulose membrane for western blotting for Foxa1, E-cadherin, and vimentin. **G. Detection of E-cadherin in cells with stable expression of miRNA-584 or/and miRNA-1290.** pcDNA6.2-GW/EmGFP-miR containing miRNA-584 or/and miRNA -1290 was transfected into SW620 colon cancer cells. Cells were separated at 24 h after transfection and screened by 5 µg/mL puromycin for 7 days. Survival clones were picked up and amplified respectively for 4 weeks. Cells were lysed and the supernatant was detected for E-cadherin. GAPDH was as controls.

Besides sustaining Erk1/2 and activating NF-κB pathways, miRNAs have hundreds of possible target genes. To identify the key target molecules of miRNA-584 and miRNA-1290, we looked for transcription factor targets common to both miRNA-584 and miRNA-1290. We first used cDNA expression profile microarrays to analyze the transcription factor profile of gastric carcinoma AGS cells. Second, we used TargetScan to screen candidate target transcription factors of miRNA-584 and miRNA-1290. Finally, the target molecules of miRNA-584 and miRNA-1290 were compared and identified (**Figure 4a**). Out of more than 1000 transcription factors, we obtained 15 possible miRNA-584 and 28 possible miRNA-1290 target transcription factors, of which *Foxa*1, *Smad*2, *Bach*1, *MITF*, and *HoxC*13 were possibly shared in common (**Figure 4b**). Among these five possible common target transcription factors, the pioneer transcription factor *Foxa*1 had higher prediction probabilities; it was also significantly down-regulated in cDNA expression profile microarrays of transiently transfected miRNA-584 into AGS cells (**Figure S5**). These results indicated *Foxa*1 as a strong candidate target molecule of both miRNA-584 and miRNA-1290.

To determine whether *Foxa*1 is a target molecule of miRNA-584 and miRNA-1290, we cloned a DNA fragment from the *Foxa*1 3′UTR into the pMIR reporter vector and then co-transfected this plasmid with miRNA-584 or miRNA-1290 into AGS cells. A luciferase activity assay showed that co-transfection of miRNA-584 or miRNA-1290 with pMIR-*Foxa*1 inhibited firefly luciferase activity by more than 90% compared with pMIR-*Foxa*1 alone (**Figure 4c**), indicating that miRNA-584 and miRNA-1290 act on *Foxa*1 mRNA. Western blot analysis showed that after transfection with either miRNA-584 or miRNA-1290, the expression of Foxa1 protein was down-regulated approximately 50% (**Figure 4d** and **Figure S6**). These results further indicate that *Foxa*1 is a target of miRNA-584 and miRNA-1290.

Silencing *Foxa*1 may interfere with the normal differentiation of gastric epithelial stem cells [27]–[31]. To determine the effects of down-regulating *Foxa*1 on cellular function, we monitored the stem cell ratio and EMT in cells undergoing *Foxa*1 silencing. Because currently available gastric cancer cell lines mostly have low levels of *Foxa*1 and E-cadherin or even do not express them, and because *H*.*pylori* is associated with the genesis of colon cancer [32], we selected SW620 colon cancer cells with high levels of Foxa1 and E-cadherin expression for this study. We infected common SW620 cells with *Foxa*1 shRNA recombinant lentiviruses, and flow cytometry revealed that the percentage of CD44^+^CD133^+^ cells significantly decreased in *Foxa*1-silenced SW620 cells, *P*<0.05 (ANOVA). Further studies on an important stem cell–associated molecule using western blot analysis showed that Bmi1 expression significantly decreased during *Foxa*1 silencing (**Figure 4e**), suggesting that silencing of *Foxa*1 promoted the transformation of stem cells into progenitor cells through down-regulation of the Bmi1 pathway. Moreover, western blot analysis showed that E-cadherin expression decreased and vimentin expression increased in these cells (**Figure 4f**). Overexpression of miRNA-584 or/and miRNA-1290 also decreased E-cadherin level (**Figure 4g**). These results suggest that *Foxa*1 is a key transcription factor and that down-regulating Foxa1 expression promotes EMT and interferes with the differentiation of stem cells.

### 5. Overexpression of miRNA-584 and miRNA-1290 Induced Intestinal Metaplasia in Knock-in Mice

**Figure 5:**
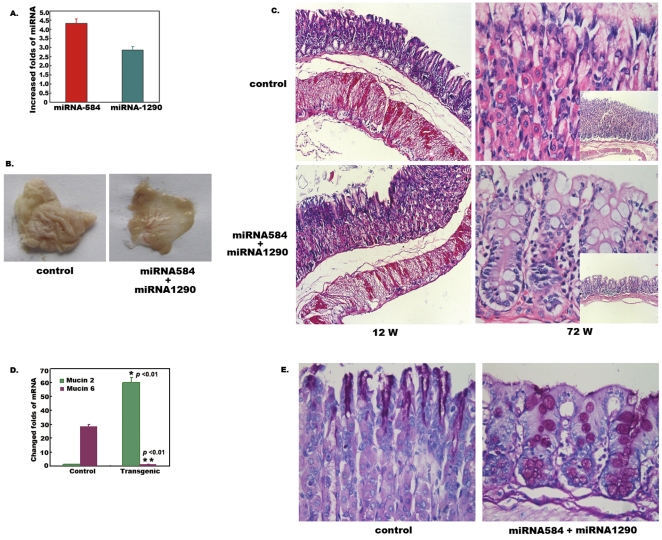
Overexpression of miRNA-584 and miRNA-1290 induced intestinal metaplasia in knock-in mice. A. Determination of miRNA-584 and miRNA-1290 in knock-in mice. Total RNA was extracted from minced tissues of mice by Trizol reagent. Real-time PCR was performed for determination of miRNA-584 and miRNA-1290. The results showed miRNA-584 and miRNA-1290 increased in knock-in mice. Data are represented as mean +/− s.e.m. **B. Morphological observation of knock-in mice.** Obvious morphological alterations were not observed at the 12th week in knock-in mice. However, gastric mucosa layers were significantly thinned and flattened, and rugal folds had disappeared at the 72nd week **C. H&E staining of gastric mucosa.** Obvious morphological alterations were not observed at the 12th week in knock-in mice. However, gastric epitheliums were replaced by intestinal metaplasia under light microscopy (×100). **D. Determination of** ***Muc*** **2 and** ***Muc*** **6.** Total RNA was extracted from minced gastric tissues of mice by Trizol reagent. Real-time PCR was performed for determination of *Muc*2 and *Muc*6. The results showed *Muc*2 was significantly increased while *Muc*6 was significantly decreased in transgenic mice (all *P* <0.01, ANOVA). Data are represented as mean +/−s.e.m. **E. PAS staining.** Gastric mucosa from transgenic mice at 72nd W were checked by PAS staining (×100). The results showed that PAS-positive cells were increased in in transgenic mice.

To explore the developmental affections of gastric epithelial stem cell with abnormal EMT and differentiation induced by miRNA-584 and miRNA-1290, we produced knock-in mice with overexpression of mature miRNA-584 and miRNA-1290. The results showed that the levels of miRNA-584 and miRNA-1290 were up-regulated about 4.32- and 2.85-fold, respectively, in knock-in mouse, as validated by Taqman miRNA assays (**Figure 5a**). Obvious morphological alterations were not observed at the 12th week in knock-in mice. However, gastric mucosa layers were significantly thinned and flattened, and rugal folds had disappeared at the 72nd week (**Figure 5b**). Gastric mucosa was completely replaced by large intestinal epithelium under microscopy (**Figure 5c**). *Muc*2, an important marker of intestinal epithelials, was significantly increased while *Muc*6, an important marker of gastric epithelials, was significantly decreased in transgenic mice (**Figure 5d**). Periodic Acid-Schiff (PAS) staining showed that PAS-positive cells were also increased in in transgenic mice (**Figure 5e**). These results demonstrated that overexpression of miRNA-584 and miRNA-1290 induced transdifferentiation of gastric epithelial cells in knock-in mice.

## Discussion

Our results show that *H*.*pylori* CagA protein can up-regulate the expression of both miRNA-584 in an NF-κB–dependent manner and miRNA-1290 in an Erk1/2-dependent manner. miRNA-584 sustained Erk1/2 activities through inhibition of PPP2a activities, and miRNA-1290 activated NF-κB through knockdown of *NKRF*. Further luciferase reporter assays and western blot revealed *Foxa*1 to be an important target of miRNA-584 and miRNA-1290. Knockdown of *Foxa*1 promoted the EMT significantly. Overexpression of miRNA-584 and miRNA-1290 induced intestinal metaplasia of gastric epithelial cells in knock-in mice. Thus, Erk1/2 activitied by CagA drived miRNA-1290 expression and subsequently miRNA-584 acivation. We speculated that different *H*.*pylori* strains owned different ability to up-regulate miRNA-1290, due to *cagA* diversity at C-terminus, in particular which from Western strains with variable number of EPIYA repeat.

The biological functions of miRNA-584 and miRNA-1290 are not clear. miRNA-584 is down-regulated in human renal clear cell carcinoma and was proposed to have tumor suppressor properties [33]. In this study, we found that miRNA-584 could inhibit Smad2 expression as shown by western blot analysis (**Figure S7**). Moreover, it could inhibit PPP2a activities, which play an important role in attenuation of the Erk1/2 pathway by direct de-phosphorylation of Erk1/2 kinase. The inhibition of Y-Box protein 1 in drug-sensitive gastric cancer cells can lead to increased miRNA-1290 expression [34], whereas no other important targets were identified. We found a strong relationship between miRNA-1290 and miRNA-584 in clinic colon cancer tissue. miRNA-1290 could repress NKRF to enhance NF-κB activities, which may be involved in the development and progression of cancer. Therefore, both miRNAs were also proposed to have oncogenic properties.

One miRNA usually has hundreds of possible target genes, making it difficult to identify the key target molecules of any miRNA. One important intersection between miRNA interference and signaling pathways is altered transcription factor levels; therefore, we looked for transcription factors targeted by both miRNA-584 and miRNA-1290. Candidate target transcription factors were identified by using TargetScan to find transcription factors targeted by both of these miRNAs in gastric carcinoma AGS cells. Out of more than 1,000 transcription factors we found 15 possibly targeted by miRNA-584 and 28 possibly targeted by miRNA-1290, with *Foxa*1, *Smad*2, *Bach*1, *MITF*, and *HoxC*13 being common to both miRNAs. Among these five possible target transcription factors, *Foxa*1 had higher context scores in both the miRNA-584 and miRNA-1290 predictions. Subsequent firefly luciferase reporter assays and western blot analysis confirmed that *Foxa*1 was knocked down by miRNA-584 and miRNA-1290. FoxA family members include Foxa1, Foxa2, and Foxa3. Foxa1 is expressed in the middle and late embryonic stages; Foxa1 expression significantly increases in embryonic cells after retinoic acid induction [35]. Foxa1 and Smad2 form a complex on the α-fetoprotein (*AFP*) promoter to promote *AFP* transcription [36]. Foxa1 is a pioneer transcription factor that can promote tissue-specific transcription and is closely linked with epigenetic signatures of tissues and cells [37]. Foxa1 is involved in the tissue development of the liver, gall bladder, lung, pancreas, intestine, and prostate. Silencing of *Foxa*1 and/or *Foxa*2 may result in abnormal development and differentiation of those tissues and cells. Silencing of *Foxa*1 increases the expression of sonic hedgehog, Foxa2, and Notch, terminates the differentiation of prostate epithelial cells, and causes abnormal phenotypes in glands; silencing of *Foxa*1 and/or *Foxa*2 causes lung epithelial dysplasia and epithelial hyperplasia. In the intestine, Foxa1 and Foxa2 are only expressed in epithelial stem cells; silencing of *Foxa*1 and/or *Foxa*2 causes goblet cell hyperplasia, alters the properties of the secretory mucins, and causes abnormal differentiation of enteroendocrine cells [27]–[31]. The functions of Foxa1 and Foxa2 are complementary; however, we found that Foxa1-positive cells were not perfectly coincident with Foxa2-positive cells in gastric mucosa by immunohistochemical staining. Foxa1-positive cells could be found in superfacial mucosa (**Figure S8**), whereas Foxa2-positive cells only appeared in the bottom of the gland.

EMT is the process of transformation from an epithelial cell phenotype to a mesenchymal phenotype [38]–[40]. EMT is an important component of the processes of cell differentiation. During the process of cell differentiation, EMT is precisely regulated in space and time. Our results indicate that although miRNA-584 up-regulates Erk1/2 activity, it can induce EMT through the inhibition of Foxa1, suggesting that the infiltrating phenotype associated with CagA may be associated with up-regulation of miRNA-584 and miRNA-1290. In addition, the percentage of stem cells in *Foxa*1-silenced SW620 cells is significantly lower and Bmi1 is also down-regulated. These findings suggest that silencing of Foxa1 may promote the transformation of stem cells into progenitor cells. Unlike RNA interference, which has only one target, miRNA-584 and miRNA-1290 have hundreds of possible target genes besides the mentioned targets above. To explore the pathogenic phenotype of gastric epithelial cells with overexpression of miRNA-584 and miRNA-1290, we observed the transition of gastric epithelial to large intestinal epithelial by producing knock-in mice, although miRNA-584 was not a conserved miRNA in mice.

Our study indicates that *H*.*pylori* CagA protein promotes EMT and interferes with cell differentiation by up-regulating miRNA-584 and miRNA-1290. Thus, the miRNA pathway is a new pathogenic mechanism of CagA. This study provides evidence that interference with miRNA pathways is one of the pathogenic mechanisms of microorganisms.

## Materials and Methods

### Ethics Statement

This research was approved by the Institutional Review Boards of Second Affiliated Hospital of Zhejiang University School of Medicine. All Animal works had been conducted according to relevant national and international guidelines.

### H.pylori and cagA/pEGFP-c1 Plasmid

*cagA*+ *H*.*pylori* and *cagA*- *H*.*pylori* strains were isolated from clinic gastric mucosa and the status of cag pathogenicity island was identified by PCR respectively. The *cagA/pEGFP-c*1 plasmid was constructed as follows: full-length *cagA* DNA was amplified by PCRs from the NCTC 11637 *H*.*pylori* strain (ATCC) and subsequently cloned into the *pEGFP-c*1 vector (BD Biosciences), and the sequence of amino acid was identical to that of the NCTC 11637 strain by DNA sequencing (protein accession number: C6K4R4).

### DNA Sequences

All DNA sequences used in this study were deposited in GenBank. *FoxA*1 accession number: U39840; *PPP*2*ca* accession number: X12646; *NKRF* accession number: AJ011812, *Smad*2 accession number: U59911; *cagA* accession number: ACS34714.1.

### Methods

#### 1. Cell culture

Gastric carcinoma AGS, SGC7901 and 293T cells were from the Cell Bank of Chinese Academy of Sciences (Shanghai, China). Cells were grown in RPMI 1640 culture medium (Gibco) supplemented with 100 U/ml penicillin, 100 µg/ml streptomycin, and 15% fetal bovine serum (FBS) (Sijiqing Biotech, China); cells were passaged every 2 to 3 days. SW620 colon cancer cells were cultured in Leibovitz’s 15 (L-15) culture medium (Gibco) supplemented with 100 U/ml penicillin, 100 µg/ml streptomycin, and 15% FBS; 293T cells were cultured in DMEM culture medium (Gibco) supplemented with 100 U/ml penicillin, 100 µg/ml streptomycin, and 15% FBS;cells were passaged every 3 to 4 days.

#### 2. Cell transfection

The standard calcium phosphate precipitation method (Clontech) was used to establish the stable CagA-expressing AGS cell line. Briefly, the culture medium of AGS cells cultured in a 35-mm culture dish was replaced with fresh culture medium 3 h before transfection. Three micrograms of *cagA/pEGFP-c*1 plasmid and 12.4 µL of 2 M CaCl_2_ were added to H_2_O to make a 100-µL solution, which was mixed thoroughly. The mixture was then added slowly into 100 µL of 2×HBS solution and placed at room temperature for 20 min. After thorough mixing, the mixture was added onto the surface of the cells. After culturing at 37°C for 12 h in a 5% CO_2_ incubator, the medium was refreshed with new culture medium. Cells were passaged 48 h after transfection at a 1∶8 ratio; 500 µg/mL G418 (Clontech) was added the next day to select transfected cells for 7 to 10 days. Transfection was validated using PCR and DNA sequencing after cell clones had grown. A control AGS cell line stably expressing the empty *pEGFP-c*1 vector was also established.

Transient transfection of cells was performed using Lipofectamine 2000 liposomes (Invitrogen) or Fugene HD (Roche) strictly according to the manufacturer’s instructions. Eight microliters of Lipofectamine 2000 liposomes or Fugene HD were added into 100 µL OPTI-MEM culture medium (Gibco) containing 2 µg plasmids. This was mixed thoroughly and placed at room temperature for 15 min. The solution was then added onto cells cultured in 35-mm culture plates and mixed thoroughly. After culturing at 37°C for 48 h in a 5% CO_2_ incubator, cells were harvested.

#### 3. MicroRNA screening

RNA was extracted from AGS cells stably expressing CagA or the empty vector using Trizol reagent (Invitrogen) and was sent to CapitalBio, Beijing, for mammalian miRNA expression profile microarray detection, which included 1024 miRNAs from mouse, rat, and human. The results were compared with those from the control group; changes that were >2-fold or <2-fold were considered significant.

#### 4. Real-time PCR

Detection of mature miRNAs was performed using the TaqMan real-time PCR method (Applied Biosystems) according to the manufacturer’s instructions. RNA was isolated from AGS cells using Trizol; after quantitation, reverse transcription was performed using 10 ng RNA and primers specific for mature miR-584 or miR-1290. The reverse transcription was run at 16°C for 30 min followed by 42°C for 30 min; the reaction was inactivated at 85°C for 5 min. The reverse transcription products were mixed with TaqMan universal PCR master mix II, and real-time PCR was performed. The PCR program was 95°C for 10 min, followed by 40 cycles of 15 s at 95°C and 60 s at 60°C.

For detection of *Muc*2 and *Muc*6, RNA was isolated from gastric tissue of transgenic mice using Trizol; after quantitation, reverse transcription was performed using 10 ng RNA and 50 µM oligodT_15_ primers. The reverse transcription was run at 37°C for 15 min followed by 85°C for 5 s; the reaction was inactivated at 85°C for 5 min. The reverse transcription products were mixed with specific primers and probes for *Muc*2 and *Muc*6, and real-time PCR (Takara) was performed. *Muc*2 top: 5′-aatggcatgc agaccaatta-3′. bottom: 5′-tcttctgcatgttcccaaac-3′. Probe: 5'Fam-caatggcactgaactgtatgccttcc -3'Tamra. *Muc*6: top: 5′-tctccactgttgctgtctcc-3′. bottom: 5′-acgaggtctga ggagctgat-3′. Probe: 5'Fam-tgca gaccacc attgcctccc-3′Tamra. The PCR program was 95°C for 30s min, followed by 40 cycles of 5 s at 95°C and 34s at 60°C.

#### 5. Construction of miR-584 and miR-1290 expression plasmids

The sequences of mature miR-584 and miR-1290 were chemically synthesized as follows. miRNA -584 top: 5′-ctagtagtttacaggtctgtggcaatactcttaaccataag aattgaaatggta-3′; bottom: 5′-agcttaccatttcaattcttatggttaagagtattgcc acagacctgtaaacta-3′. miRNA-1290 top: 5′-ctagtaacgtggtccaagattcaaaaa tcctattgatagtggccattta-3′; bottom: 5′-agcttaaatggccactatcaataggattt ttgaatcttggaccacgtta-3′. After denaturation at 95°C for 4 min, 4 µL of 10 nM oligos were ligated with 2 µL of 5 ng/µL linearized pcDNA6.2-GW/EmGFP-miR plasmid (Invitrogen) using T4 DNA ligase at room temperature for 1 h. The ligation mixture was transformed into TOP10 cells (Invitrogen), and colonies were selected by 50 µg/mL spectinomycin. Colonies were amplified to extract plasmids for DNA sequence identification.

#### 6. Detection of pMIR-*Foxa*1 report luciferase activity

DNA fragments from the *Foxa*1 3′UTR that might interact with miR-584 and miRNA-1290 were chemically synthesized. miRNA-584 top: 5′-ctagtatagcaat attcttggagattgataaccatagcattaatacgcccatta-3′; bottom: 5′-agcttaatg ggcgtattaatgctatggttatcaatctccaagaatattgctata-3′. miRNA-1290 top: 5′-ctagtcaaaccgtcaacagcataataaaatcccaacaactatttttatttcattttt ca-3′; bottom: 5′-agcttgaaaaatgaaataaaaatagttgttgggattttattatgctgtt gacggtttga-3′. DNA fragments were re-natured at 90°C for 3 min followed by 37°C for 60 min. The dsOligos were then ligated into the SpeI/HindIII sites in the MCS of linearized pMIR-report luciferase plasmid (3∶1) (Ambion) using T4 ligase at room temperature for 1 h. The ligation mixture was then transformed into *E. coli* DH5α. Colonies were selected by 100 µg/mL ampicillin. Colonies were then amplified to extract plasmid for DNA sequence identification. To assess pMIR luciferase activity, recombinant pMIR-*Foxa*1 plasmids were co-transfected with pcDNA6.2-GW/EmGFP-miR plasmids containing miRNA-584 or miRNA-1290 into gastric carcinoma AGS cells for 48 h. Cells were harvested, and luciferase activity was determined by dual-luminescence reporter assay (Promega).

#### 7. Construction of promoter of miR-584 and miR-1290 luciferase reporter plasmids

The promoter sequences of miR-584 and miR-1290 were amplified by the following primers: miRNA-584 top: 5′-agtctgtggtaccggtggggaagtc agagatga-3′; bottom: 5′-agtctgtaagctttccctgaagcaaccttcttg-3′. miRNA-1290 top: 5′-agtctgtg gtacccccaacctttctcagagcag-3′; bottom: 5′-agtctgtaagctttggcattgggt ctttctttc-3′. After PCR, the products were digested by Kpn I and Hind III and ligated with linearized pGL3 plasmid (Promega) using T4 DNA ligase at 22°C overnight. The ligation mixture was transformed into *E. coli* DH5α cells, and colonies were selected by 100 µg/mL ampicillin. Colonies were amplified to extract plasmids for DNA sequence identification.

#### 8. Western blotting

Cells (1×10^6^) were harvested and washed with phosphate-buffered saline (PBS) twice. They were then lysed with 350 µL radio-immunoprecipitation assay (RIPA) buffer containing 1% NP-40, 0.25% sodium deoxycholate, 5 mM DL-Dithiothreitol (DTT), and 1× protease inhibitor cocktail on ice for 5 min. The cell lysate was centrifuged at 13,000 g for 10 min, and the supernatant was mixed with an equal volume of sample buffer. After 10% sodium dodecyl sulfate–polyacrylamide gel electrophoresis (SDS-PAGE), proteins were transferred onto a nitrocellulose membrane. After blocking with 5% non-fat milk at room temperature for 30 min, the membrane was washed three times and then incubated with diluted primary antibodies: Foxa1 antibody (Abcam) and antibodies to Bmi1, E-cadherin, vimentin, PPP2ca and NKRF (Epitopmics) at room temperature for 2 h or 4°C overnight. After five washes, the membrane was incubated with a 1∶2,000 dilution of horseradish peroxidase (HRP)-labeled goat anti-rabbit antibody at room temperature for 1 h. The membrane was developed using enhanced chemiluminescence (ECL) (Cellsignal Biotech) after five washes.

#### 9. Recombinant lentivirus infection

Five thousand SW620 colon cancer cells were cultured in a 12-well plate at 37°C overnight in a 5% CO_2_ incubator. The culture medium was changed to fresh medium containing 8 µg/mL polybrene (Sigma) the next day, and 25 µL of recombinant lentivirus solution (Santa Crutz) were added for 24 h at 37°C in a 5% CO_2_ incubator. The culture medium was then changed, and the cells were digested and passaged at a 1∶8 ratio when the cells were 2/3 confluent. Puromycin (5 µg/mL) (Clontech) was added the next day to select for lentivirus-infected cells for 7 to 10 days. After colonies had grown, western blot analysis was performed for identification. An empty vector was transfected as a control.

#### 10. Flow cytometry

SW620 colon cancer cells infected with recombinant lentivirus were digested with 0.25% trypsin, and cells were then collected by centrifugation at 1,500 g for 5 min. The cells were washed twice with PBS containing 2% FBS and blocked in buffer on ice for 10 min. After centrifugation at 2,000 g for 3 min, the cells were resuspended at 1×10^6^ per 100 µL of buffer. Labeling antibodies (15 µL APC-CD44 and 15 µL PE-CD133) (Miltenyi) were added at 4°C for 30 min in the dark. After centrifugation, the cells were washed with buffer twice and resuspended in 500 µL buffer. They were then analyzed using a FACSAria cell sorter (BD Biosciences). A blank control and an isotype control were included.

#### 11. Production of miRNA-584 and miRNA-1290 knock-in mice

The production of mice with overexpression of mature miRNA-584 and miRNA-1290 was performed by Shanghai Innovation Biotechnology Co. Ltd. Briefly, mature sequences of miRNA-584 and miRNA-1290 from pcDNA6.2-GW/EmGFP-miR plasmids were cloned into BLOCK-iT™ Lentiviral Pol II miR RNAi expression vectors (Invitrogen) and transfected into FT293 cells (Invitrogen) to produce recombinant lentivirus. Subsequently densed lentivirus particles were injected into oosphere cells of C57BL6 mice for the production of mice with overexpression of mature miRNA-584 and miRNA-1290. Finally, the levels of miRNA-584 and miRNA-1290 were validated by Taqman miRNA assays.

#### 12. Infection model

1×10^5^/well AGS cells were seeded into a 12-well plate at 37°C overnight in a 5% CO_2_ incubator for 24 h. Subsequently cells were infected with *H*.*pylori* including NCTC11637 and 9 *cagA*– isolates for additional 7 h (MOI≈50) respectively, and TaqMan real-time PCR was performed.

## Supporting Information

Figure S1 **Putative Elk-1** **binding sites in the promoter regions of miRNA-584 and miRNA-1290.**
(DOC)

Figure S2 **Relationship between miRNA-584 and miRNA-1290 in clinic tumor. tissues.** Total RNA from 9 pairs of human primary colon cancer tissues were isolated and real-time PCR was performed for miRNA-84 and miRNA-290. The results showed a strong relationship between miRNA-584 and miRNA-1290 in clinic tumor tissue samples.
(DOC)

Figure S3 **Putative c-Rel** **binding sites in the promoter regions of miRNA-584.**
(DOC)

Figure S4 **Putative binding sites of miRNA-584 in 3′-UTR of PPP2a.**
(DOC)

Figure S5 ***Foxa*** **1 expression is lower in AGS cells transiently expressing miRNA-584.** The plasmid pcDNA6.2-GW/EmGFP-miR containing mature miRNA-584 was transiently transfected into AGS cells with Lipofectamine 2000. Total RNA was extracted, and human cDNA genechip scanning was performed. Differential genes were identified by real-time PCR. The results show that *Foxa*1 expression is lower in AGS cells transiently expressing miRNA-584.
(DOC)

Figure S6 **Normalized protein expression of** **Foxa1.** Densitometric analysis of bands corresponding to Foxa1 and GAPDH was performed using Quantity-One software (Bio-Rad). The expression of Foxa1 protein was down-regulated about 50% after transfection of miRNA-584 or miRNA-1290 (*P* <0.01 and *P* <0.05, ANOVA. Data are represented as mean +/– s.e.m.). Protein expression was normalized to GAPDH.
(DOC)

Figure S7 **miRNA-584 decreases the expression of Smad2.** pcDNA6.2-GW/EmGFP -miR containing the mature miRNA-584 DNA sequence was transiently transfected into AGS cells with Lipofectamine 2000 for 48 h. Cells were harvested and lysed. The supernatant of the lysate was subject to SDS-PAGE, and proteins were transferred onto a nitrocellulose membrane. After blocking with non-fat milk, the membrane was incubated with anti-Smad2 antibody and diluted HRP-labeled goat anti-rabbit antibody. Finally. the membrane was developed using ECL substrate.
(DOC)

Figure S8 **Expression of FoxA1 in gastric epithelial cells.** Gastric mucosa from a patient with H.*pylori* infection was sliced after fixation with formalin. The slices were analyzed by immunohistochemical staining after antigen retrieval.
(DOC)
