# Supplementary figures and images for: MicroRNAs Up-Regulated by CagA of Helicobacter pylori Induce Intestinal Metaplasia of Gastric Epithelial Cells
Source: PLoS One. 2012 Apr 20;7(4):e35147. doi: 10.1371/journal.pone.0035147 (PMC3335061; doi:10.1371/journal.pone.0035147)

**Figure S1.**

**
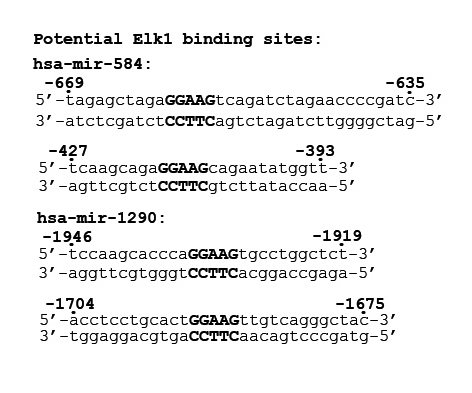
**

Supplement: Figure S1 — Putative Elk-1 binding sites in the promoter regions of miRNA-584 and miRNA-1290. (DOC) [file pone.0035147.s001.doc]

**Figure S2.**

**
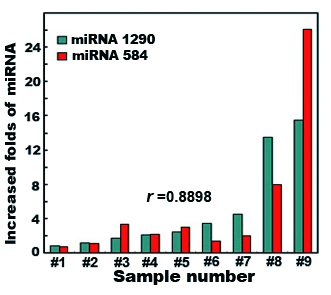
**

Supplement: Figure S2 — Relationship between miRNA-584 and miRNA-1290 in clinic tumor. tissues. Total RNA from 9 pairs of human primary colon cancer tissues were isolated and real-time PCR was performed for miRNA-84 and miRNA-290. The results showed a strong relationship between miRNA-584 and miRNA-1290 in clinic tumor tissue samples. (DOC) [file pone.0035147.s002.doc]

**Figure S3.**

**
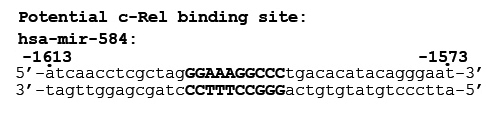
**

Supplement: Figure S3 — Putative c-Rel binding sites in the promoter regions of miRNA-584. (DOC) [file pone.0035147.s003.doc]

**Figure S4.**

**
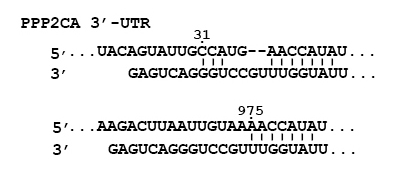
**

Supplement: Figure S4 — Putative binding sites of miRNA-584 in 3′-UTR of PPP2a. (DOC) [file pone.0035147.s004.doc]

**Figure S5.**

**
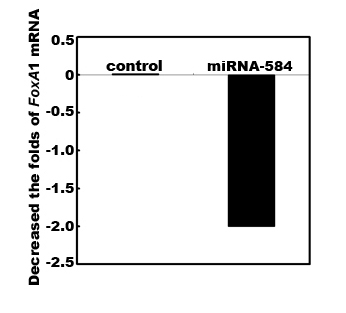
**

Supplement: Figure S5 — Foxa 1 expression is lower in AGS cells transiently expressing miRNA-584. The plasmid pcDNA6.2-GW/EmGFP-miR containing mature miRNA-584 was transiently transfected into AGS cells with Lipofectamine 2000. Total RNA was extracted, and human cDNA genechip scanning was performed. Differential genes were identified by real-time PCR. The results show that Foxa1 expression is lower in AGS cells transiently expressing miRNA-584. (DOC) [file pone.0035147.s005.doc]

**Figure S6.**

**
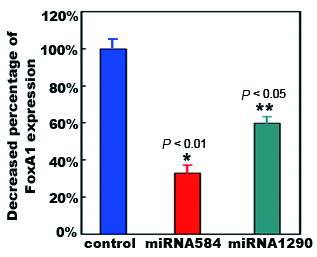
**

Supplement: Figure S6 — Normalized protein expression of Foxa1. Densitometric analysis of bands corresponding to Foxa1 and GAPDH was performed using Quantity-One software (Bio-Rad). The expression of Foxa1 protein was down-regulated about 50% after transfection of miRNA-584 or miRNA-1290 (P <0.01 and P <0.05, ANOVA. Data are represented as mean +/– s.e.m.). Protein expression was normalized to GAPDH. (DOC) [file pone.0035147.s006.doc]

**Figure S7.**

**
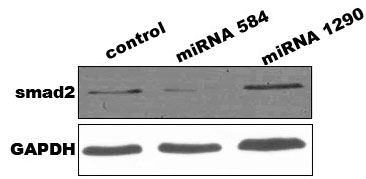
**

Supplement: Figure S7 — miRNA-584 decreases the expression of Smad2. pcDNA6.2-GW/EmGFP -miR containing the mature miRNA-584 DNA sequence was transiently transfected into AGS cells with Lipofectamine 2000 for 48 h. Cells were harvested and lysed. The supernatant of the lysate was subject to SDS-PAGE, and proteins were transferred onto a nitrocellulose membrane. After blocking with non-fat milk, the membrane was incubated with anti-Smad2 antibody and diluted HRP-labeled goat anti-rabbit antibody. Finally. the membrane was developed using ECL substrate. (DOC) [file pone.0035147.s007.doc]

**Figure S8.**


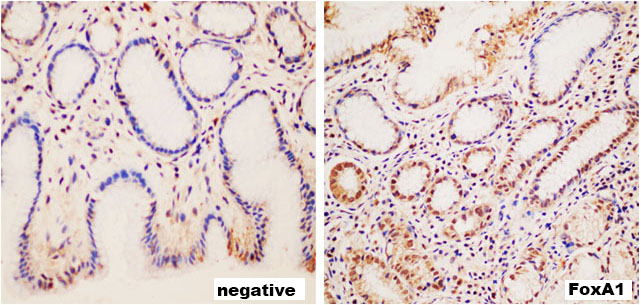

Supplement: Figure S8 — Expression of FoxA1 in gastric epithelial cells. Gastric mucosa from a patient with H.pylori infection was sliced after fixation with formalin. The slices were analyzed by immunohistochemical staining after antigen retrieval. (DOC) [file pone.0035147.s008.doc]
